# Environmental Enrichment Blunts Ethanol Consumption after Restraint Stress in C57BL/6 Mice

**DOI:** 10.1371/journal.pone.0170317

**Published:** 2017-01-20

**Authors:** Priscila Marianno, Karina Possa Abrahao, Rosana Camarini

**Affiliations:** 1 Departamento de Farmacologia, Instituto de Ciências Biomédicas, Universidade de São Paulo, São Paulo, São Paulo, Brazil; 2 Laboratory for Integrative Neuroscience, Division of Intramural Clinical and Biological Research, National Institute on Alcohol Abuse and Alcoholism, National Institutes of Health, Bethesda, Maryland, United States of America; Oregon Health and Science University, UNITED STATES

## Abstract

Elevated alcohol intake after abstinence is a key feature of the addiction process. Some studies have shown that environmental enrichment (EE) affects ethanol intake and other reinforcing effects. However, different EE protocols may vary in their ability to influence alcohol consumption and stress-induced intake. The present study evaluated whether short (3 h) or continuous (24 h) EE protocols affect ethanol consumption after periods of withdrawal. Mice were challenged with stressful stimuli (24 h isolation and restraint stress) to evaluate the effects of stress on drinking. Male C57BL/6 mice were subjected to a two-bottle choice drinking-in-the-dark paradigm for 15 days (20% ethanol and water, 2 h/day, acquisition phase). Control mice were housed under standard conditions (SC). In the first experiment, one group of mice was housed under EE conditions 24 h/day (EE24h). In the second experiment, the exposure to EE was reduced to 3 h/day (EE3h). After the acquisition phase, the animals were deprived of ethanol for 6 days, followed by 2 h ethanol access once a week. Animals were tested in the elevated plus maze (EPM) during ethanol withdrawal. During the last 2 weeks, the mice were exposed to 24 h ethanol access. A 1-h restraint stress test was performed immediately before the last ethanol exposure. EE24h but not EE3h increased anxiety-like behavior during withdrawal compared to controls. Neither EE24h nor EE3h affected ethanol consumption during the 2 h weekly exposure periods. However, EE24h and EE3h mice that were exposed to acute restraint stress consumed less ethanol than controls during a 24 h ethanol access. These results showed that EE reduces alcohol intake after an acute restraint stress.

## Introduction

Alcohol addiction is a complex psychiatric disorder strongly influenced by environmental factors [1]. Animal models can mimic one or more aspects of addiction. Attention has been given to animal models that can elicit high levels of alcohol consumption and preference [2]. High ethanol intake, in which the animal may overcome the initial aversive taste of ethanol and drink sufficient quantities to achieve a state of intoxication [3], can be evaluated in self-administration models. In one such model, two bottles are presented to the animals: one containing tap water and another containing an alcohol solution [4]. Previous studies have shown that alcohol consumption in the two-bottle choice paradigm can be further increased after periods of ethanol deprivation [5, 6, 7, 8], which may mimic some aspects of repeated withdrawals, persistent craving and relapse in humans [9]. This phenomenon is called alcohol deprivation effect (ADE). In these experimental models, inbred C57BL/6 mice have been widely used because they present a predisposition to voluntarily consume significant quantities of alcohol to the point of behavioral intoxication [10].

Considering the strong influence of environmental factors on alcohol addiction, there has been increased interest in models that control the environment, either positively or negatively. The introduction of objects that allow animals to voluntarily exercise, play, and interact with different toys provides the animals with a positive environment that can help them better cope with stress. These environmental manipulations are called environmental enrichment (EE) [11, 12], which is able to alter novelty-seeking behavior, impulsivity, and anxiety-like behavior [13, 14, 15, 16].

EE decreases psychostimulant self-administration and drug-seeking behavior [17, 18]. Mice living in enriched conditions during periods of abstinence exhibited reductions of behavioral sensitization to cocaine, cocaine-induced reinstatement and conditioned place preference (CPP) [19]. Previously, we found that EE also prevented the development and expression of ethanol-induced behavioral sensitization [20], meanwhile the influence of EE on ethanol consumption appears to be inconsistent. While Rockman et al. [21] showed that rats reared under EE conditions exhibited an increase in voluntary ethanol consumption, Deehan et al. [22] found decreased ethanol consumption, preference, and motivation to obtain ethanol in the operant self-administration paradigm in alcohol-preferring rats reared under similar EE conditions. Other studies showed that EE can reduce ethanol consumption and ethanol-induced CPP [23, 24], as well as protect adolescent single-housed mice from increasing ethanol intake during adulthood [25].

While positive environmental conditions, as the EE, may reduce the reinforcing effects of drugs, stressful conditions tends to increase these effects and play an important role in determining the vulnerability to addiction [26]. Epidemiological studies have shown that individuals who presented high levels of alcohol intake experienced high levels of stress [27]. Stressful events can also enhance the reinstatement of drug-seeking behavior after extinction [28]. However, no consensus has been reached concerning the way in which stress influences ethanol consumption (for review see Becker et al. [29]).

There are evidences that EE can reduce drug-seeking behavior induced by cues, stress and stress-induced disruptions of the hypothalamic-pituitary-adrenal axis [30, 31, 32]. It also confers resilience to animals that are exposed to social defeat stress [33]. Thus, we hypothesized that EE might prevent an increase of ethanol drinking induced by stress. In the present study, we evaluated whether short (3 h) or continuous (24 h) exposure to EE affect ethanol consumption after repeated withdrawal cycles and after an acute stress situation.

## Materials and Methods

### Animals

Fifty adult male C57BL/6 mice, 8–10 weeks old, were housed in groups of five or six in large transparent polycarbonate cages (42 cm length × 28 cm width × 21.5 cm height) in a room with controlled temperature (22 ± 1°C) and a 12 h/12 h light/dark cycle (lights off at 9:00 AM). The animals had free access to food and water and were acclimatized to the reverse cycle for at least 2 weeks before the experiments. Red incandescent lights were used so that the investigators could handle the mice during the dark phase. All experimental procedures were approved by the Ethical Committee for Animal Use of the Instituto de Ciências Biomédicas, Universidade de Sao Paulo, Brazil (protocol no. 143).

### Drinking in the dark

The drinking in the dark (DID) protocol was used to assess voluntary ethanol intake and modified from previous described methods [34]. The animals had free access to water throughout the procedure to avoid ethanol consumption motivated by water deprivation and thirst. Three hours after beginning of the dark phase, the animals were single-housed with free access to two bottles (two-bottle choice paradigm): one bottle contained ethanol (95% v/v; Labsynth, Diadema, SP, Brazil) diluted in tap water at 20% (v/v), and the other bottle contained tap water only. The mice were allowed to drink both solutions for 2 h. The bottles were then removed, and the mice were returned to their home cages. The bottles were weighed before and immediately after the consumption sessions. Differences in the weights of the bottles were converted to volumes of ethanol and water solutions ingested. Ethanol consumption in g/kg was calculated considering the ethanol’s density, the concentration of the ethanol solution, the amount of solution consumed and the body weight of each subject. The solutions were changed daily, and the positions of the bottles were switched to obviate possible side preferences. During the entire DID procedure, one cage with two bottles was used as a control for leakage from the bottles, caused by handling or evaporation. The volume lost in these control bottles was subtracted from the volume of ethanol or water drunk by each mice. Spontaneous leakage was avoided by using nozzles with two balls (Ancare Corp., Bellmore, NY, USA).

### Environmental enrichment

Various plastic objects and toys, such as houses, pipes, ramps, ladders, balls and metal or plastic running wheels (one running wheel and one house per cage was maintained during the entire experiment) were added in the home cage of EE group. The objects (Cinoteck, São Paulo, SP, Brazil; Innovive, San Diego, CA, USA; Kaytee, Chilton, WI, USA) were purchased in pet shops. Their positions were changed three times per week to maintain environmental novelty. Exposure to EE began the day following the acquisition phase and continued throughout the experiment.

### Elevated plus maze

A single test session in the EPM was performed during the dark phase of the light/dark cycle to be consistent with the timing of the DID procedure. The apparatus comprised two open arms (33.5 cm × 7 cm, with 0.5 cm high ledges) and two closed arms (33.5 cm × 7 cm, with 20 cm high walls) in a plus configuration that extended from a common central platform (10 cm × 10 cm). The apparatus was elevated 50 cm above the floor. The animals were taken to the experimental room 15 min before testing for adaptation to the new environment. The mice were placed in the central platform facing an open arm and the behaviors were evaluated in a single 5 min session. Each trial was recorded with a digital camera. The recorded data included entries into and time spent on the open arms (percentage of total recording time), total entries into both the open and closed arms, latency to the first open arm entry, and stretched attend posture. The first entry was recorded when the animal placed all four paws in a specific arm.

### Restraint stress

On the last day of the consumption protocol, the mice were placed in an individual ventilated polypropylene tube (diameter of 3 cm and length of 11.5 cm) for 1 h before re-exposure to the DID test. The procedure was realized in the dark and the animals started the ethanol consumption immediately after the restraint stress procedure.

### Experimental design

#### Experiment 1: Exposure to EE 24 h/day

**Fig 1:**
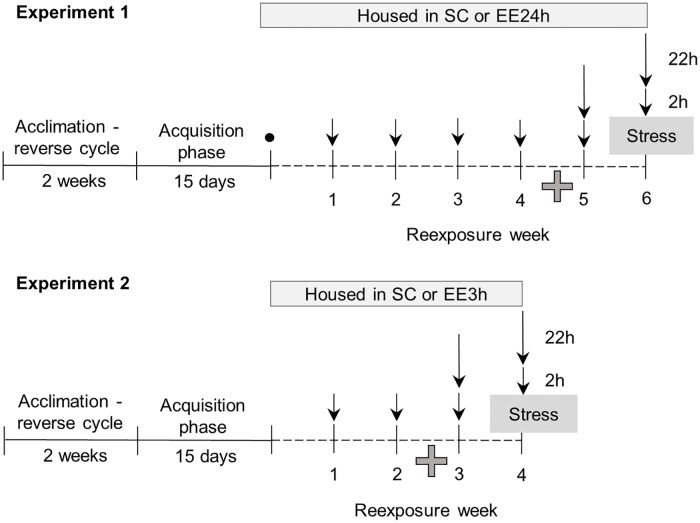
Experimental design. The short arrows represent weekly exposure to the two-bottles choice paradigm (20% ethanol and water) for 2 h. The long arrows represent re-exposures for additional 22 h. Cross = elevated plus maze test. • = BEC measurement.

The main purpose of this experiment was to evaluate whether continuous EE (24 h/day) could alter ethanol consumption after periods of withdrawal and after a stressful situation. The experimental design is shown in Fig 1. Twenty-one mice were exposed to the drinking-in-the-dark (DID) paradigm for 15 days for the acquisition and stabilization of ethanol consumption. To facilitate the escalation of ethanol consumption, we included three periods of ethanol deprivation (2 days off). Immediately after the last consumption session (day 15), approximately 20 μl blood samples were collected from the submandibular vein using a 5.0 mm lancet [35]. Blood was collected in a heparinized tube and centrifuged at 2,000 x *g* for 10 min at 4°C to separate plasma and then stored at -20°C until the time of the assay. The plasma was used to quantify blood ethanol concentrations (BECs) using an enzymatic assay and Analox GL6 Multiassay Analyser (Analox Instruments, Lunenburg, MA, USA). Blood ethanol concentrations are expressed as milligrams of ethanol per milliliter of blood (mg/ml). Following the acquisition phase, the animals were randomly distributed into two groups: standard housing conditions (SC; *n* = 10) and EE for 24 h/day (EE24h; *n* = 11). After six days of ethanol deprivation, the mice were offered two bottles containing either 20% ethanol or tap water for 2 h only, once a week, for 4 weeks. The mice were tested in the elevated plus maze (EPM) on week 5. On weeks 5 and 6, mice were given 24 h free access to ethanol and the consumption was measured 2 and 24 h from the start of drinking. Next, in order to evaluate if restraint stress alters ethanol consumption, mice were subjected to a 1-h restraint stress session before being given 24-h free access to ethanol at week 6 of the experiment. Ethanol consumption can decrease immediately after exposure to a restraint stress [36], so we also measured the consumption after 2 and 24 h of ethanol drinking.

#### Experiment 2: Exposure to EE 3 h/day

This experiment followed similar experimental design (Fig 1), with the exception that mice were exposed to EE for 3 h/day instead of 24 h/day. Twenty animals were subjected to the acquisition phase and then randomly allocated to two groups: control (SC, *n* = 10) and EE3h (*n* = 10). In this experimental procedure, the objects were placed in the cages immediately after lights were off (9:00 AM) and then removed after 3 h (12:00 PM). Cages of the control mice were manipulated by the experimenter as a control for the disturbances caused by manipulating the objects in the EE3h cages. Considering that in Experiment 1 we observed no significant differences in ethanol consumption during the first five re-exposures to ethanol, we limited the re-exposures to 4 weeks in Experiment 2. During re-exposures at weeks 3 and 4, intake was measured twice (i.e., at the end of the first 2 h and at 24 h after the onset of consumption). The EPM test was performed on the 3rd week, and the animals were exposed to 1-h restraint stress on the 4th week, immediately before the ethanol consumption test. Considering the differences in ethanol consumption between the EE and SC groups during the 24 h of ethanol access after stress exposure in Experiment 1, we measured plasma corticosterone levels in this experiment. Blood samples were collected after euthanasia, and plasma corticosterone levels were determined in duplicate using a Corticosterone EIA Kit (Cayman Chemical, Ann Arbor, MI, USA) according to the manufacturer’s instructions.

A supplementary experiment to investigate whether stress alters ethanol intake was performed following the same protocol as described in Experiment 2, with the exception that the mice (*n* = 9) had access only to the ethanol bottle during the 2h re-exposure tests. Water bottles were not offered (S1 Appendix).

### Statistical analysis

Ethanol and water consumption during the acquisition phase were analyzed using one-way repeated-measure analysis of variance (ANOVA), with day as the repeated measure. Ethanol intake (g/kg) over 2 and 24 h was analyzed using a two-way repeated-measures ANOVA, with housing condition as the between-subjects factor and week as the within-subjects factor. Significant interactions were followed by the Newman-Keuls *post hoc* test. In order to consider inter-individual differences among mice and the variation of ethanol intake throughout the acquisition phase, the re-exposure consumption data were transformed into a percent change from the mean of the last 5 days of the acquisition phase for each mouse (i.e., 100% represented the average consumption during the last 5 days of the acquisition phase for each animal and we determined the percent change from this baseline, as done in [37]). The EPM data and corticosterone levels were analyzed using Student’s *t*-test when the data had a normal distribution or the Mann-Whitney test for nonparametric data. Pearson test was used to determine correlation between ethanol consumption and BECs. The results were analyzed using Statistica 7.0 software, and the graphical data were generated using GraphPad Prism 5.0. The data are expressed as mean ± SEM. Values of *p* < 0.05 were considered statistically significant.

## Results

### Experiment 1: Exposure to EE 24 h/day

**Fig 2:**
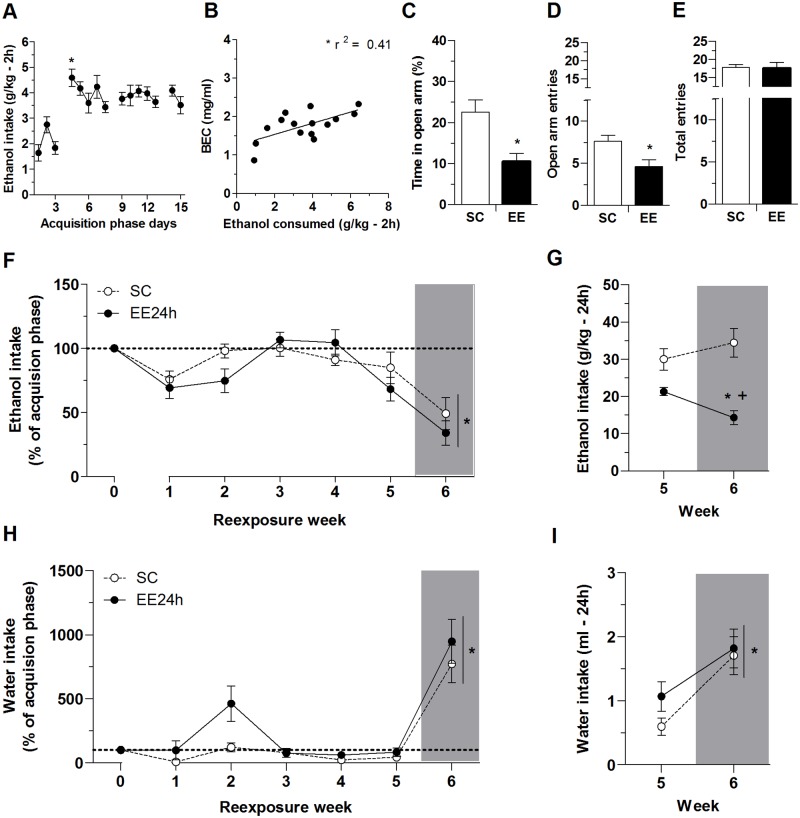
Experiment 1. (A) Ethanol consumption for 2 h/day during 15 days of acquisition phase. (B) Blood ethanol concentration (mg/ml) plotted against ethanol consumed (g/kg) during 2 h of ethanol access. Positive correlation was found between the amount of ethanol consumed and BECs (*r*^*2*^ = 0.41, *p* < 0.05). (C-E) Elevated plus maze test: (C) percent time on open arms, (D) number of open arm entries, and (E) total number of arm entries. (F) Ethanol intake during 2 h access to two-bottle choice once a week. Ethanol intake was converted to percent of basal ethanol consumption. Basal ethanol consumption was calculated by averaging the absolute consumption of the last 5 days of acquisition phase, and converted as 100% (week 0). (G) Ethanol intake (g/kg) during 24 h of access to two-bottle choice on weeks 5 and 6. (H) Water intake (ml) during 2 h access to two-bottle choice. Water intake was converted to percent of basal water consumption. Basal water consumption was calculated by averaging the absolute consumption of the last 5 days of acquisition phase, and converted as 100% (week 0). (I) Water intake (ml) during 24 h. Gray bars represent the measures of intake after exposure to the restraint stress procedure. The data are expressed as mean ± SEM, except in B. *p < 0.05, compared with days 1, 2, and 3 in A, compared with prior re-exposure weeks in F, H, and I, and compared with the EE group on the previous week without stress in G; ^+^*p* < 0.05, compared with SC group.

The analysis of the 15-day acquisition phase revealed a significant effect of day, with an increase in ethanol intake following the first ethanol deprivation relative to the three previous days (*F*_14,280_ = 11.05, *p* < 0.05). By the end of the acquisition phase, the mice reached stable levels of ethanol consumption, i.e., no differences were found among the last 5 days (Fig 2A). Blood samples were taken after the 2 h drinking session on day 15 of the acquisition phase, and 15 samples contained sufficient plasma for BEC analysis. The results revealed mean BECs of 1.79 ± 0.10 mg/ml and mean ethanol consumption of 3.41 ± 0.47 g/kg, which are consistent with intoxicating levels. A significant positive correlation was found between the levels of ethanol intake and BECs (*r*^*2*^ = 0.41, *p* < 0.05; Fig 2B).

EE24h elicited anxiety-like behavior (Fig 2C and 2D), reflected by less time spent on the open arms (*t* = 3.50, *p* < 0.05) and fewer entries into the open arm (*t* = 2.78, *p* < 0.05) when compared with the SC group (Fig 2C and 2D, respectively). The total number of arm entries did not differ between groups (*t* = 0.08), suggesting no difference in locomotor activity between mice from SC and EE24h groups (Fig 2E). We also observed no differences in the latency to the first open arm entry (Mann-Whitney test, *U* = 42) or stretched attend posture (*t* = 0.24; data not shown).

Fig 2F shows the percentage of ethanol intake in relation to the acquisition phase. The repeated measure ANOVA showed no effects of housing condition on ethanol intake (*F*_1,19_ = 1.42, *p* > 0.05) and no housing condition × week interaction (*F*_5,95_ = 2.00, *p* > 0.05). While EE did not alter 2-h ethanol consumption during the five re-exposures (weeks 1–5), a significant decrease in 2-h ethanol intake was observed in both the SC and EE groups after restraint stress on week 6 (week factor: *F*_5,95_ = 13.24, *p* < 0.05).

Fig 2H shows the water intake in relation to the acquisition phase. The ANOVA did not show significant effects of housing condition (*F*_1,19_ = 3.28, *p* > 0.05) or housing condition × week interaction (*F*_5,95_ = 1.38, *p* > 0.05). However, after a restraint stress protocol, mice consumed more water during the first 2 hours of exposure to the 2-bottles compared to the previous weeks (week factor: *F*_5,95_ = 29.57, *p* < 0.05).

Ethanol and water consumption was also measured at the end of 24 h of free access to both solutions on weeks 5 and 6 (Fig 2G and 2I). Regarding ethanol consumption (Fig 2G), the ANOVA found as significant the housing condition factor (*F*_1,19_ = 20.01, *p* < 0.05) and the housing condition × week factor interaction (*F*_1,19_ = 13.90, *p* < 0.05). No differences were found in ethanol intake over the 24 h ethanol access between the SC and EE groups in week 5. After the restraint stress, ethanol consumption of control mice was similar to that observed in the previous week, but the EE group exhibited lower ethanol consumption in week 6 compared with the consumption in week 5. In addition, the EE group consumed less ethanol than the SC group after the stress exposure. Regarding water consumption, the ANOVA revealed a significant effect of week (*F*_1,19_ = 24.58, *p* < 0.05) but no effect of housing condition (*F*_1,19_ = 0.79) and no housing condition × week interaction (*F*_1,19_ = 0.81) (Fig 2I). Both groups exhibited higher water intake on week 6 compared with week 5, likely attributable to a robust increase in water consumption during the 2 h right after the stress exposure.

### Experiment 2: Exposure to EE 3 h/day

**Fig 3:**
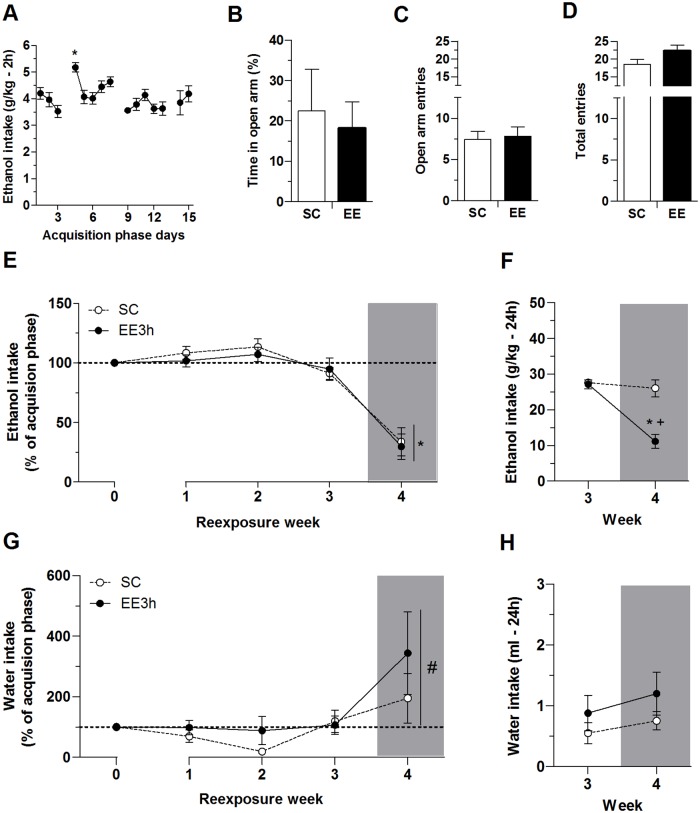
Experiment 2. (A) Ethanol consumption for 2 h/day during 15 days of acquisition phase. (B-D) Elevated plus maze test: (B) percent time on open arms, (C) number of open arm entries, and (D) total number of arm entries. (E) Ethanol intake during 2 h access to two-bottle choice once per week. Ethanol intake calculations followed the same criteria as described for the first experiment. (F) Ethanol intake (g/kg) during 24 h of access to two-bottle choice on weeks 3 and 4. (G) Water intake during 2 h access to two-bottle choice. Water intake calculations followed the same criteria as described for the first experiment. (H) Water intake (ml) during 24h. Gray bars represent the measures of intake after exposure to the restraint stress procedure. The data are expressed as mean ± SEM. *p < 0.05, compared with day 3 in A, compared with the other re-exposure weeks in E, and compared with the EE group on the previous week without stress in F; ^+^*p* < 0.05, compared with SC group; ^#^*p* < 0.05, compared with weeks 2 and 3.

Consistent with the data from Experiment 1, the analysis of the 15-day acquisition phase revealed an increase in ethanol consumption after the first ethanol deprivation period when compared to the previous day, with a significant effect of day (*F*_14,266_ = 3.13, *p* < 0.05). During the last 5 days, mice reached stable levels of ethanol consumption and no differences were found among these days (Fig 3A). Exposure to EE for 3 h/day (EE3h) did not induce anxiety-like behavior or alter locomotor activity (Fig 3B and 3C). No differences were found between the SC and EE3h groups in the time spent in the open arms (*t* = 1.02), number of open arm entries (*t* = - 0.27), or total number of arm entries in the EPM (*t* = - 1.77; Fig 3D). The latency to the first open arm entry (*t* = - 1.63) and number of stretched attend posture (*t* = 1.75) did not differ between groups (data not shown). Together with the previous experiments, these results indicate that only continuous exposure to EE induced an anxiety-like effect in mice.

Similar to Experiment 1, EE3h did not alter the consumption of either ethanol or water during the weekly 2 h DID sessions. After exposure to acute restraint stress (week 4), both groups exhibited a decrease in ethanol consumption (effect of week: *F*_3,54_ = 37.99, *p* < 0.05) and increase in water consumption (effect of week: *F*_3,54_ = 2.85, *p* < 0.05) compared with weeks 2 and 3 (Fig 3E and 3G, respectively). In Fig 3E, the ANOVA revealed no effect of housing condition on ethanol intake (*F*_1,18_ = 1.45, *p* < 0.05) and no housing condition × week interaction (*F*_3,54_ = 0.23, *p* > 0.05). In Fig 3G, the ANOVA revealed no effect of housing condition on water intake (*F*_1,18_ = 0.52; *p* > 0.05) and no housing condition × week interaction (*F*_3,54_ = 0.75, *p* > 0.05).

Following the stress exposure on week 4, ethanol and water consumption was also measured after 24 h (see Fig 3F and 3H, respectively). In Fig 3F, the ANOVA revealed significant effects of housing condition (*F*_1,18_ = 17.94, *p* < 0.05) and week (*F*_1,18_ = 30.95, *p* < 0.05) and a housing condition × week interaction (*F*_1,18_ = 20.10, *p* < 0.05). Similar to Experiment 1, no differences were found in ethanol intake over 24 h between the SC and EE groups on week 3. In addition, the SC group exhibited no differences in ethanol intake between weeks 3 and 4. The EE3h group though consumed less ethanol after acute stress in week 4 compared to week 3 and less ethanol than the SC group in week 4 (Fig 3F). Regarding the water consumption, no effects of housing condition (*F*_1,18_ = 1.16, *p* > 0.05) or weeks (*F*_1,18_ = 1.07, *p* > 0.05) on water consumption were found, with no housing condition × week interaction (*F*_1,18_ = 0.81, *p* > 0.05; Fig 3H).

Environmental-related differences in corticosterone levels were investigated at the end of this experiment. The EE and SC groups did not differ in plasma corticosterone levels after 24 h ethanol intake (*t* = -1.49; data not shown).

## Discussion

The present study showed that continuous, but not intermittent, EE increased anxiety-like behavior in mice. In addition, restraint stress decreased ethanol consumption during the first 2 h access to the 2-bottle choice regardless the environmental condition. However, mice living in a continuous EE (24 h/day) or restricted EE (3 h/day) consumed less alcohol over a 24 h period after an acute stressful stimulus compared to mice living in a standard condition.

In this study, mice reached intoxicant levels of BECs when exposed to intermittent two-bottles choice DID test. It is important to consider that although the DID protocol has been previously shown to result in pharmacologically relevant BECs (> 1 mg/ml) in 2 h sessions [34], the supply of water bottles can reduce BECs by approximately 40% [38]. On the other hand, short ethanol exposure (3 days), followed by a short deprivation period (2 days), consistently increased ethanol intake [6, 7] which may have contributed to the fast escalation of ethanol drinking observed in this study.

We found that continuous EE exposure increased anxiety-like behavior. Previous studies reported contradictory results of EE effects on anxiety, which showed decreased, unchanged or even increased anxiety-like behavior in animals that lived in an enriched environment (for review, see [12, 39]). The variability in EE and EPM protocols may be responsible for the disparate results [12, 40]. In the present study, the duration of daily exposure to EE influenced the expression of anxiety-like behavior in mice. The continuous novelty and complexity of the stimuli that were offered to these mice could have caused some degree of stress in mice that were previously habituated to living in an “impoverished” environment (i.e., standard laboratory conditions). This effect was not observed when environmental changes were temporary (3 h/day). The present study also found no differences in the total number of entries into the open and closed arms of the EPM between groups, although a decrease in locomotor activity has been reported in EE mice using this type of apparatus [16].

In both humans and animals, one common symptom of ethanol withdrawal is the manifestation of anxiety disorders, due to activation of anxiety-related circuitry. It is known that ethanol withdrawal-induced anxiety may facilitate relapse to ethanol drinking in consequence of its negative reinforcing properties [41]. We hypothesized that EE might reduce ethanol relapse after abstinence by decreasing ethanol withdrawal-induced anxiety-like behavior. In animal models, a number of studies have demonstrated the anxiolytic effects of EE, although some studies have shown no effect or even increased anxiety (see [12] for review). However, in our study the housing condition did not affect ethanol drinking. First, one could rationalize that the drinking/withdrawal protocol that was used in the present study did not generate pronounced “anxiety” in abstinent animals, which might explain the lack of differences in ethanol intake between the two housing conditions. According to Cox et al. [2], the DID paradigm combined with periods of withdrawal increases ethanol consumption and preference without increasing anxiety-like behavior during withdrawal. Thus, this model not necessarily accompanies all hallmarks of alcohol dependence. Second, EE24h induced an anxiogenic rather than an anxiolytic effect. EE is comparable to repeated mild stress and may be considered as “positive stress,” or “eustress” [39, 42, 43], which differs from negative stressors [44]. Considering EE as a potential “eustressor”, this would justify its anxiogenic effect detected by the plus-maze. EE could predispose the mice to a mild anxiety sufficient to counteract a more severe anxiety condition (induced by restraint stress). In fact, EE exerts a protective effect against anxiety induced by diverse stressors [31, 45]. This hypothesis would explain the reduced alcohol intake in EE mice only after stress exposure. Previous studies suggested that EE affects addiction-related behaviors only when the levels of stress are sufficiently high to cause pronounced “anxiety”. For example, EE did not reduce cocaine-induced reinstatement in a self-administration model [30], unless it is induced by yohimbine, a pharmacological stressor that causes anxiety-like responses in animals [46]. In the present study, EE mice exhibited lower ethanol intake compared with SC mice when exposed to a combination of 1 h restraint stress plus 24-h social isolation. Social isolation has been used as a model of posttraumatic stress disorder to recapitulate several symptoms of this psychiatric disorder [47]. Indeed, acute restraint stress induces anxiety-like behavior in rats [48], which can be minimized by EE [49].

Twenty-four hours of free access to ethanol uncovered differences between groups in both experiments, likely resulting from exposure to stress. Animals that were housed under EE conditions exhibited a reduction of ethanol consumption compared with the previous week without stress exposure. However, ethanol consumption in the SC groups during these two re-exposures did not differ. Notably, both groups exhibited a decrease in ethanol consumption 2 h after stress, what is in agreement with [36]. The stress type may influence ethanol-drinking behavior. In Cozzoli et al [36] restraint stress decreased ethanol consumption during 2h-access in DID for at least 2 days in mice. On the other hand, 15 hours after immobilization stress (1 h) rats self-administered significantly more ethanol at least over 12 days [50]. In the present study, over the next 22 h, the control group exhibited a recovery in the amount of alcohol consumed comparable to the amount they had consumed in the previous week, reaching similar levels of intake as those that were reached during the week without restraint stress. By measuring the ethanol intake over 24 h it was possible to verify the lasting consequences of the stress not only its immediate effects.

Over 24 h access to ethanol EE groups exhibited lower ethanol consumption compared with the previous week without stress and also compared with SC mice. Although SC and EE groups showed differing levels of alcohol intake following restraint stress in Week 6 relative to Week 5, such result may reflect a maintenance of the reduced intake in the EE group but not in the SC group after stress. Thus, an alternative hypothesis is that EE made these mice susceptible to the restraint stress-induced decrease in alcohol drinking. To support this hypothesis, EE24h increased anxiety in those mice as revealed by the elevated plus maze test, suggesting higher susceptibility to the effects of stressful stimuli. However, it is also important to emphasize that EE3h induced similar reduced ethanol intake, but did not increase anxiety-like behavior or altered the corticosterone levels measured at the end of the 24 hours of ethanol exposure. In previous study, we have demonstrated that EE prevented stress-induced increase in glucocorticoid receptor signaling in the basolateral amygdala of rats, without preventing the rise in corticosterone serum levels after acute restraint stress [49].

Importantly, no differences in the first 2 h of ethanol intake were observed after acute stress exposure between SC and EE. This was associated with a significant decrease in ethanol intake and an increase in water intake after the restraint stress protocol. It is possible that restraint stress caused a considerable dehydration. A previous study reported a marked increase in total urine production and decrease in osmolality after immobilization stress in rats [51]. The dysregulation of fluid balance can trigger regulatory mechanisms that maintain the homeostasis of bodily fluids, such as hypothalamic mechanisms of thirst control [52], inducing a substantial increase in water intake immediately after restraint stress exposure. The changes in water intake could be affecting the ethanol intake. However, when the present experiment was performed without the water bottle, ethanol intake also decreased during the first 2 h after acute stress exposure (S1 Fig). These data suggest that the decrease in ethanol intake may not be only a consequence of the increase in water intake but rather also a behavioral reaction to stress.

In conclusion, we raised the hypothesis that this positive stress induced by EE could help the animals better cope with aversive situations, thus reducing stress reactivity [33, 53] and attenuating ethanol consumption. In fact, EE appears to attenuate the responses that are generated by various stressors, such as electric shocks, predator exposure, and restraint stress (for review, see [31]), thereby exerting a protective effect in adverse situations. However, we cannot discard the hypothesis that EE increased the susceptibility of those mice to the effects of stressful stimuli and extended the low ethanol intake over a longer period. Further studies should be conducted to clarify these questions. Based on the present results, we conclude that mice exposed to EE showed attenuated alcohol intake only after an acute restraint stress.

## Supporting Information

S1 Appendix Supplementary Experiment. Experimental procedures and results.
(DOCX)

S1 Fig Ethanol intake during 2h access to one-bottle containing 20% ethanol after restraint stress. Gray bars represent the measure of ethanol intake after exposure to 1-h restraint stress. Ethanol intake was converted to percent of basal ethanol consumption. Basal ethanol consumption was calculated by averaging the absolute consumption of the last 5 days of acquisition phase, and converted as 100% = week 0, and then all values of ethanol intake were converted to percentage of this basal consumption. Upon the fourth re-exposure after stress, the mice exhibited lower ethanol consumption compared with the previous weeks (one-way repeated-measures ANOVA; effect of week: *F*_3,24_ = 12.56, *p* < 0.05). The data are expressed as mean ± SEM. *p < 0.05, compared with the previous re-exposures.
(TIF)
