# Supplementary material for: Environmental Enrichment Blunts Ethanol Consumption after Restraint Stress in C57BL/6 Mice
Source: PLoS One. 2017 Jan 20;12(1):e0170317. doi: 10.1371/journal.pone.0170317 (PMC5249154; doi:10.1371/journal.pone.0170317)
Supplement: S1 Appendix — Experimental procedures and results. (DOCX) [file pone.0170317.s001.docx]

**S1 Appendix. Supplementary Experiment.** Experimental procedures and results.

We investigated whether the decrease in ethanol intake observed after acute restraint stress could be associated with the pronounced increase of water consumption induced by the stress. In this experiment, mice (*n* = 9) were exposed to a single bottle containing 20% ethanol during the whole experiment. Ethanol intake was measured during 2 h access to two-bottle choice once per week (DID protocol).

One way-ANOVA revealed a decrease in ethanol consumption over 2 h after exposure to restraint stress compared with prior exposures (*F*_3,24_ = 12.56, *p* < 0.05), suggesting that stress decreased ethanol consumption even when the water bottle was not available.
