# Supplementary figures and images for: Environmental Enrichment Blunts Ethanol Consumption after Restraint Stress in C57BL/6 Mice
Source: PLoS One. 2017 Jan 20;12(1):e0170317. doi: 10.1371/journal.pone.0170317 (PMC5249154; doi:10.1371/journal.pone.0170317)

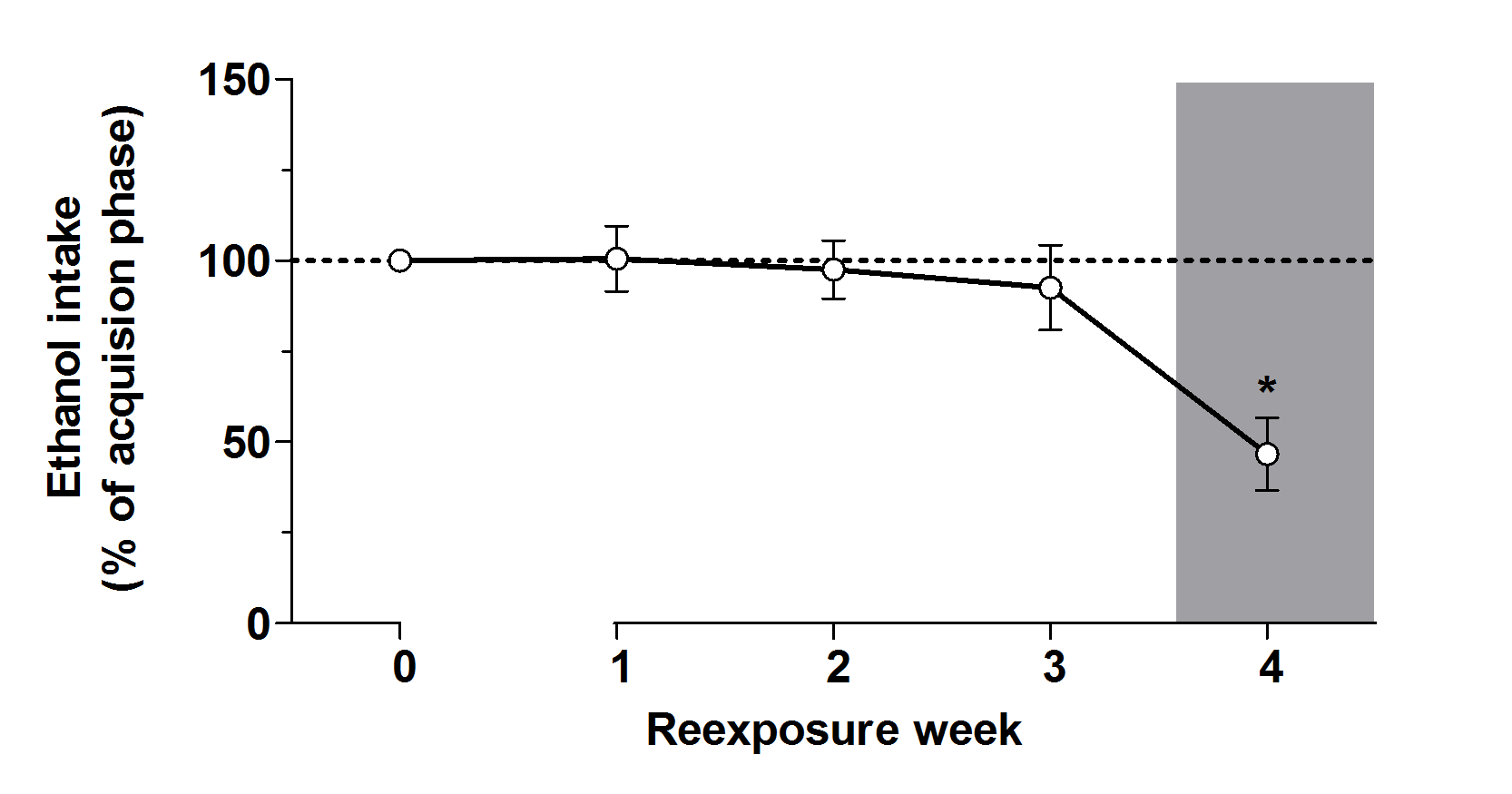

Supplement: S1 Fig — Gray bars represent the measure of ethanol intake after exposure to 1-h restraint stress. Ethanol intake was converted to percent of basal ethanol consumption. Basal ethanol consumption was calculated by averaging the absolute consumption of the last 5 days of acquisition phase, and converted as 100% = week 0, and then all values of ethanol intake were converted to percentage of this basal consumption. Upon the fourth re-exposure after stress, the mice exhibited lower ethanol consumption compared with the previous weeks (one-way repeated-measures ANOVA; effect of week: F3,24 = 12.56, p < 0.05). The data are expressed as mean ± SEM. *p < 0.05, compared with the previous re-exposures. (TIF) [file pone.0170317.s002.tif]
